# A Neuromorphic Architecture for Object Recognition and Motion Anticipation Using Burst-STDP

**DOI:** 10.1371/journal.pone.0036958

**Published:** 2012-05-15

**Authors:** Andrew Nere, Umberto Olcese, David Balduzzi, Giulio Tononi

**Affiliations:** 1 Department of Electrical and Computer Engineering, University of Wisconsin-Madison, Madison, Wisconsin, United States of America; 2 Department of Psychiatry, University of Wisconsin-Madison, Madison, Wisconsin, United States of America; 3 Department of Neuroscience and Brain Technologies, Istituto Italiano di Tecnologia, Genova, Italy; 4 Department of Empirical Inference, Max Planck Institute for Intelligent Systems, Tubingen, Germany; The University of Plymouth, United Kingdom

## Abstract

In this work we investigate the possibilities offered by a minimal framework of artificial spiking neurons to be deployed *in silico*. Here we introduce a hierarchical network architecture of spiking neurons which learns to recognize moving objects in a visual environment and determine the correct motor output for each object. These tasks are learned through both supervised and unsupervised spike timing dependent plasticity (STDP). STDP is responsible for the strengthening (or weakening) of synapses in relation to pre- and post-synaptic spike times and has been described as a Hebbian paradigm taking place both *in vitro* and *in vivo*. We utilize a variation of STDP learning, called burst-STDP, which is based on the notion that, since spikes are expensive in terms of energy consumption, then strong bursting activity carries more information than single (sparse) spikes. Furthermore, this learning algorithm takes advantage of homeostatic renormalization, which has been hypothesized to promote memory consolidation during NREM sleep. Using this learning rule, we design a spiking neural network architecture capable of object recognition, motion detection, attention towards important objects, and motor control outputs. We demonstrate the abilities of our design in a simple environment with distractor objects, multiple objects moving concurrently, and in the presence of noise. Most importantly, we show how this neural network is capable of performing these tasks using a simple leaky-integrate-and-fire (LIF) neuron model with binary synapses, making it fully compatible with state-of-the-art digital neuromorphic hardware designs. As such, the building blocks and learning rules presented in this paper appear promising for scalable fully neuromorphic systems to be implemented in hardware chips.

## Introduction

The primate visual cortex exemplifies the brain's unique ability to extract and integrate vast amounts of sensory stimuli into meaningful categorizations. Within the visual cortex, multiple pathways exist for processing submodalities such as color, object recognition, and motion detection. Even more impressive is the brain's ability to combine and integrate such pathways, processing complex visual environments on the order of tens to hundreds of milliseconds [1]–[5]. Feedback connections extend the abilities of the visual cortex allowing for attention, anticipation, and prediction in spite of noisy or complicated visual scenery [6], [7]. Furthermore, the other various sensory and motor areas of the primate neocortex similarly show crossmodal processing and integration. These features of the primate neocortex, which are vital for an animal's decision making in its environment, are implemented via a single basic computational module, the spiking neuronal cell.

Because the brain is able to use such a simple functional processing unit for inherently complicated and diverse tasks, it is no wonder that computer designers have an interest in emulating many of its properties in computing hardware. Furthermore, the low-power and event-driven nature of neurons provides all the more reason for chip designers to investigate these biologically inspired computing systems [8], [9]. However, for digital neurons to rival the energy efficiency of their biological counterparts, various design decisions must be made to approximate the attributes of a spiking neuronal cell. Digital optimizations such as binary synapses, linear (or at best, piecewise-linear) operations, and sparse connectivity are clearly optimal choices with digital CMOS technology.

In this paper, we investigate a simplified model of the visual cortex in the context of digital neuromorphic hardware constraints. Our objectives are to 1) test the performance of a recently proposed biologically-inspired learning paradigm in an environment with noise and distractors and 2) show that a trained network, utilizing only the set of parameters available in a digital hardware, is able to sufficiently replicate some of the important features of the visual cortex. Our goal is therefore not to develop a model able to outperform existing state of the art models of the visual system, but to investigate the prospects offered by a hardware implementation of a fully neuromorphic architecture; while we focus on developing and testing a minimal system as a proof of concept, our goal is to develop an architecture to be eventually scaled to larger sizes in order to cope with more complex tasks and environments.

To achieve these objectives, we first introduce a hierarchically organized network of spiking neurons which learns to both recognize moving objects and determine the correct motor control output for a particular object. This biologically inspired neural network learns these tasks through a combination of supervised and unsupervised spike timing dependent plasticity (STDP) learning rules. STDP is a Hebbian learning paradigm [10]–[12] which has been shown to be responsible for the strengthening (or weakening) of synapses between neurons in relation to pre- and postsynaptic spike times, both *in vitro* and *in vivo*. In our model, we employ a recently proposed plasticity paradigm called burst-STDP [13] which modifies synaptic strength based on correlating pre- and postsynaptic bursting activity. Such a learning paradigm is based on the concept that spiking activity is expensive in terms of energy consumption [14]. Burst (rare) events must therefore convey more information that single spikes (which are more common). Burst-STDP exploits this principle by linking the magnitude of plastic change to the level of burstiness of the neurons. Additionally, this algorithm also takes advantage of homeostatic renormalization of synaptic strength, a mechanism which has been hypothesized to take place during sleep and promote memory consolidation [15], [16]. Furthermore, such a mechanism can drastically improve the signal to noise ratio in a network of neurons, as well as prime synapses for subsequent learning epochs. Finally, an important aspect of the homeostatic renormalization, as will be described, is that it promotes binary synapse convergence in the neural network.

Through learning via burst-STDP and homeostatic renormalization, we then demonstrate that our neural network is capable of object recognition, motion detection, attention towards important objects, and motor control outputs. After an epoch of offline learning, the neuron parameters adhere to their digital approximation, most importantly including binary synapses. We demonstrate the abilities of this network in a simple environment by varying the number of objects and the noise levels in a visual environment. The results show that the network architecture robustly exhibits the correct motor output in spite of such obstacles. Furthermore, we show how such behavior can be learned by a network constructed of less than 1000 simple LIF neurons, utilizing only a very small number of configurable and learned parameters, matching those present in the aforementioned neuromorphic hardware. The framework we investigate is therefore extremely simple when compared to many of the complex models present in the literature. Given the hardware-driven constraints described in this paper, we focus on developing a model able to cope with an array of simple tasks, rather than one large, complicated problem. As we will discuss, showing that such tasks can be learned and then captured by binary representations of spiking neurons is an important contribution step which highlights the abilities of low-power spiking neuromorphic hardware on silicon, paving the way for the implementation of this novel technology in robotics.

### Related work

Several attempts have been made to design neurally inspired networks able to reproduce the performance of the primate visual cortex in analyzing visual scenes. Hierarchical feedforward convolutional networks have proven to be quite successful at object recognition and represent a reference point for all works on bio-inspired object recognition. Other successful approaches such as scale-invariant feature transform (SIFT, [17]) and its evolutions are not bio-inspired and have not been considered in this work.

Models such as HMAX [18], the Neocognitron [19] on which HMAX is based, and their extensions [20], [21] have shown impressive performance at categorizing natural images, despite some recently demonstrated limitations [22] that we will later discuss. What is even more impressive about these models is that they are directly inspired by the organization of the primate visual cortex. These hierarchical models achieve their visual recognition power by alternating simple cells (S), which respond maximally for a preferred input, and complex cells (C), which provide translation invariance by using a max pooling operation over a population of simple cells. The lowest level of the HMAX model consists of Gabor filters which optimally respond to a particularly oriented edge, which is similar to the V1 area in the visual cortex. The upper layers of the HMAX model combine lower level features to achieve translation invariant recognition of images, similarly to the organization of the IT in the visual cortex [23].

In Masquelier and Thorpe's model [24], the same HMAX framework is extended to spiking neurons operating in the temporal domain. During each presentation of a training image, each neuron is able to fire at most once, with a spike latency corresponding to a neuron's selectivity for a given input. This model uses unsupervised single-spike STDP to facilitate learning between the lower level complex cells (C1) and upper level simple cells (S2). As a result, their design is able to robustly recognize images based on learned features. This model can also be considered more biologically inspired, as much of the model uses spiking neurons as the basic building block. However, the neurons in this recognition model can be described as “memoryless”, since each neuron's membrane potential is reset upon each presentation of an image. By contrast, our design utilizes LIF neurons which can maintain a membrane potential across multiple time steps, and our results will show how such a design feature contributes significantly to noise tolerance and improved decision making. A similar approach–employing non-memoryless neurons–has recently been introduced in a model of early visual areas [25], showing that orientation selectivity can spontaneously emerge thanks to the properties of STDP.

Recently, Perez-Carrasco et al. have proposed a convolutional neural network design which uses event-based computation for the vision problem, as opposed to the more traditional frame-by-frame processing used by the majority of visual system models [26]. The authors show not only that the computation is functionally equivalent, but also argue that such an approach is more biologically motivated and potentially better performing, as visual sensing and processing can now almost entirely overlap. Finally, the authors speculate that up-and-coming hardware will be based on address-event representation (AER) convolution chips, and show how their algorithm scales well with such proposed AER hardware.

Several works have also focused on bio-inspired models for motion detection. One approach–which we followed in our work–is based on the HMAX framework [27], [28] and has been shown to perform successfully on standard datasets. Other bio-inspired approaches have been able to mimic the visual stream from V1 to the medial superior temporal area (MST) [29]–[32]. Other works focused instead on modeling the properties of areas MT (medio temporal) and MST, obtaining models able to cope with complex problems, such as apertures and occlusions, with a biomimetic approach [33]–[38]. A full comparison of the various models is out of the scope of this work. Our choice was in fact based on the advantages of employing a similar architecture for both the shape classification and motion detection modules, in view of possible expansions of the network to cope with a larger and more complex environment.

The integration of multiple cortical areas has also been addressed in previous work [39]. This computer model simulates three visual streams for processing form, color, and motion. While this architecture demonstrates the interactions between several functionally segregated visual areas, it relies on a phase variable to relay the short-term temporal correlations between these areas. The network proposed here performs such integrations using an energy efficient burst-STDP learning algorithm in combination with much simpler LIF neurons.

Importantly, the entire model proposed in this paper has been designed using only LIF spiking neurons, which are among the simplest models of spiking neurons [40] and thus the most likely candidate for realization in a hardware implementation. Several hardware implementations of spiking neurons have been developed over the past years (see [41]–[44]) with much focus on optimizing communication between neurons and reducing power consumption. Our aim here is to create a minimal neural model, in terms of neurons, architecture, and plasticity mechanisms, that still shows a robust learned response for visual system tasks. With this goal in mind, this paper focuses on combining an energy efficient burst-STDP learning paradigm, a homeostatic renormalization process that performs memory consolidation and converges synapses to binary values, and simple LIF neurons that are more easily captured in digital neuromorphic hardware designs.

### Hardware constraints

In this section, we consider the design details and implications of a fabricated neuromorphic hardware, which is the hardware architecture on which we plan to deploy the neural network presented here (as well as future neural models). International Business Machine Corporation (IBM) has implemented two Neurosynaptic Cores [8], [9] for the DARPA SyNAPSE project. The goal of the SyNAPSE project is to create a system capable of interpreting real-time inputs at a biologically realistic clock rate. IBM's neuromorphic design seeks to model millions of neurons and to rival the brain in terms of area and power consumption. IBM has described one 256 neuron Neurosynaptic Core with 1024×256 programmable binary synapses [8], and another 256 neuron Neurosynaptic Core with 256×256 plastic binary synapses [9]. For the purposes of this paper, we assume that the binary synapses are programmed offline; hence we do not consider the online learning as proposed by [9]. Furthermore, these hardware neurons operate at a biologically realistic clock rate of 1kHz. Each neuron integrates spikes over one dendrite line, or column of the SRAM crossbar, and outputs spikes on an axon.

As outlined in [8], these LIF neurons utilize a very small number of configurable parameters to reduce area and power consumption. Table 1 shows these parameters and their ranges of values, as well as the number of bits necessary to capture these configurable parameters. The core is structured so that 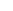 axons connect to 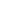 neurons via a 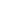 × 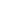 SRAM crossbar, and the synaptic connection between axon 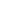 and neuron 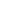 is indicated by 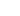. In this architecture, each neuron includes a wire for its dendritic tree and a wire for its axon. The dendritic wire contacts with N axons (from N other neurons), receiving spikes from presynaptic neurons. Each axon connects with M dendrites, thus allowing a neuron to propagate its own spikes to downstream neurons. At each time step, the activity vector 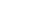 of the axons must be integrated by the neurons on the chip, and the membrane of each neuron leaks by 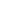. Each axon is assigned one type (excitatory, inhibitory, etc.) via the 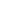 configurable parameter. Finally, 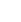 indicates the synaptic multiplier between axon 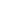 and neuron 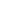. Because of the architectural decisions that went into the Neurosynaptic Core, each spike produced by these hardware neurons consumes only 45pJ, making it quite energy efficient.

**Table 1:** The settable parameters and their corresponding bit sizes for the LIF neurons implemented in digital neuromorphic hardware [8], [9].

Name	Description	Range	Bit size
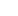	Connection vector for neuron I's dendrite	0,1	256 [9]
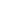	Synapse Value 0	−256 to 255	9 [8]
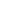	Synapse Value 1	−256 to 255	9 [8]
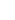	Synapse Value 2	−256 to 255	9 [8]
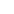	Linear Membrane Leak	−256 to 255	9 [8]
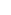	Firing Threshold	1 to 256	8 [8]
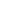	Output Axon Type	0,1,2	2 [8]

These parameters include an output axon type, three synaptic multipliers, a linear membrane leak, firing threshold, and a binary vector for synapse strengths.

For each neuron on the Neurosynaptic Core, the membrane potential of neuron *i* is updated on spiking events using:
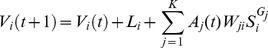
(1)


Each spike produced by a presynaptic neuron is integrated by the postsynaptic neurons in the following cycle. When a neuron produces a spike, its voltage is reset to 0.

This type of neuromorphic hardware strongly motivates the goals of our neural network design. Such hardware is attractive not only because it is low-power, but also because the underlying structure of the Neurosynaptic Core architecture much more accurately depicts the brain than does a traditional von Neumann processor. However, in order to take advantage of such hardware, we explicitly state the constraints of our neural network model to ensure compatibility with the Neurosynaptic Core:

• Binary synapses (trained offline).
• Linear membrane leak neurons.
• Reset voltage  = 0 V.
• Two axon types (excitatory, inhibitory).
• Two synaptic multipliers per neuron (S+ for excitatory, S- for inhibitory).
• Timestep  = 1ms (compatibility with 1kHz operating frequency).

This work seeks to show how burst-STDP learning and homeostatic renormalization can shape a neural network compatible with this type of hardware solution. Even though learning is performed offline, our results demonstrate how neuron models with the above mentioned parameters and constraints, can still robustly perform interesting visual system tasks.

## Methods

### An STDP Based Learning Algorithm

In this section we describe our implementation of the the burst-STDP learning algorithm, for which a more detailed description and rationale can be found in in [13]. This is a Hebbian plasticity paradigm which has been described as an effective learning rule observed in experiments both *in vitro* and *in vivo*.

**Figure 1:**
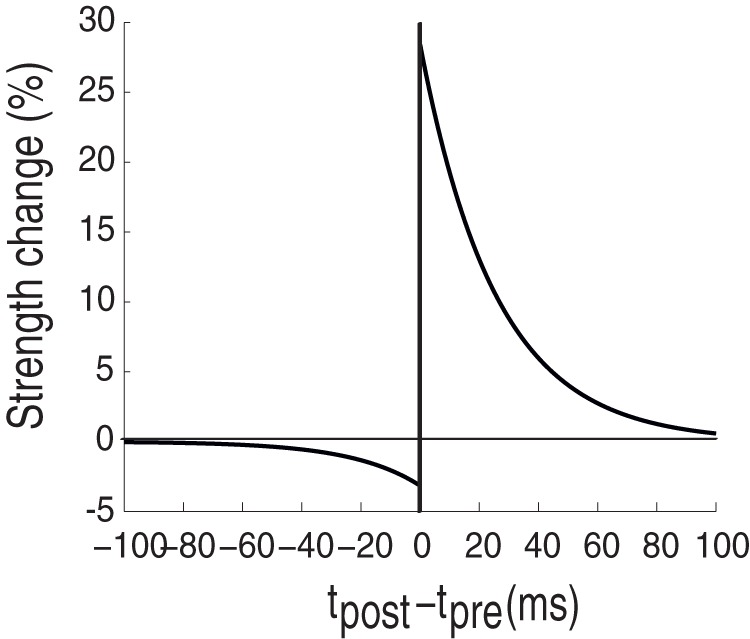
Basic STDP rule. It has been found experimentally that the strength of synaptic change is controlled by the timing between pre- and post-synaptic spikes. Here, the magnitude is estimated as the temporal relation of the post-synaptic spike to the pre-synaptic spike (origin). When the post-synaptic spike follows the pre-synaptic one, the synapse is potentiated, otherwise depressed. Moreover, the closer the two spikes are, the greater the potentiation/depression is.

In the STDP framework, plastic changes will occur on a single synapses if post- and pre-synaptic spikes both fall within an STDP timing window. If a pre-synaptic spike is followed by a post-synaptic one within the timing window, potentiation will occur. Conversely, if the post-synaptic spike is followed by the pre-synaptic, the connection will be depressed. Figure 1 shows an implementation of an STDP learning rule and demonstrates how the magnitude of strength change depends on the delay between the two spikes: the smaller the delay, the greater the change [45], [46].

Another experimentally derived property of STDP is weight-dependency, according to which the magnitude of plastic events is inversely related to the initial strength of a connection. In [46], the authors consider that no upper bound should be placed on synaptic strength, as persistent potentiation will lead a connection to a saturating value. If such an upper bound was placed on synaptic strength, all potentiated synapses would converge on the same upper bound. While not imposing a maximum synaptic weight does not necessarily mean synapses will reach infinite values, the strengths they may reach could be too large to be considered biologically plausible.

Finally, the parameters of STDP are dependent on the preceding pre-synaptic spiking history. Thus, each pre-synaptic spike allows the increase in intra-cellular calcium concentration [Ca], flowing through NMDA receptors (NMDAR). In particular, low levels of intra-cellular [Ca] favor synaptic depression, while high levels promote potentiation [47], [48]. Thus, strong pre-synaptic firing would increase intra-cellular [Ca] and favor potentiation. This phenomenon has been successfully implemented in computational models to perform STDP learning, based not only on pre- and post-synaptic spike timing, but also on firing history [49], [50].

Burst-STDP has been developed as a variant of STDP able to incorporate the fact that spikes, being energetically expensive, must be parsimoniously generated by neurons. The greater the number of spikes, therefore, the greater the saliency of the message a neuron communicates. From this it has been inferred that bursts of spikes–carrying a lot of information–should have a primary role in plastic events. Burst-STDP exploits this fact by relating plastic changes not only to the relative timing between pre- and post-synaptic events, but also on their magnitude, i.e. their level of “burstiness”.

In the following subsections we will present the three main characteristics of our learning algorithm: 1) an unsupervised burst-STDP paradigm for learning stimulus features based on their persistence on the retina, 2) reward gating for linking network responses to particular rewarded stimuli and 3) homeostatic renormalization for balancing synaptic strength.

#### Burst-STDP

As outlined in the previous sections, we modeled the unsupervised learning algorithm based on the theoretical considerations presented in [13]. Briefly, it is assumed that neurons communicate the importance of their outputs by modulating their firing rate over a certain window of time. Given that most of neurons' energy consumption is devoted to signaling [51], it is reasonable to accept that the brain as a whole should try to minimize the number of spikes necessary to convey information. Thus a neuron with a high output firing rate must be signaling a relevant event. Burst-STDP exploits the observation that spikes are expensive by modulating the magnitude of plastic change in accordance with the level of “burstiness” of pre- and post-synaptic spike trains within a certain time frame. In particular, a connection will be reinforced if a pre-synaptic burst is followed by a post-synaptic one. In a computational framework it would therefore be necessary to define and keep track of pre- and post-synaptic burstiness traces, and to implement a function relating burtiness levels to changes in synaptic strength. For our purposes–i.e. a hardware implementation of burst-STDP–we developed a simplified version of this plasticity paradigm, which requires fewer computations and has more limited memory requirements.

Burstiness is defined as a memory trace of spiking activity; the trace will decay with time, and will be increased if a spike occurs. In detail, we measure the level of pre-synaptic burstiness of each connection as:
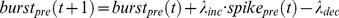
(2)


Here 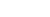 is the pre-synaptic burstiness trace and 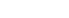 is 1 if the pre-synatic neuron fired at time t, and 0 otherwise. 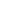 defines the increment of the burst trace on every spike, set to 0.4, and 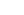 defines the decay in the burst trace at every time step, set to 0.05. Although no upper bound was placed on 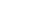, neurons and network parameters were such that we never measured any divergence in its value, i.e. the network did not show “epileptic” activity. A lower bound set at 0 was instead placed on 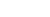, so that it could not decrease indefinitely. Instead of computing post-synaptic burstiness, we simply applied the plastic rule for each post-synaptic spike, thus implicitly giving more relevance to high levels of post-synaptic activity:
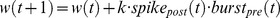
(3)


Here 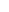 represents the learning rate. As was stated, future work will focus on deploying the entire network architecture *in silico*, complete with the burst-STDP learning rule. Hence, this simplification makes implementing a learning rule in hardware more feasible, since it can simply be modeled as a leaky trace of spiking activity and the plastic process is called at each post-synaptic spike. For the original formulation of burst-STDP [13], instead, it would be necessary to keep track of changes in burstiness across time, thus increasing the storage burden. We must point out that, in its current form, our algorithm may lead to results similar to “all-to-all spikes” STDP, as reported for example in [52], with differences being the absence of depression and a linearly rather than exponentially decreasing weight update. The similarities, however, are limited to the outcomes of the two approaches, since the rationales behind them are clearly different.

Previous research work has evidenced that learning is obtained predominantly via long-term potentiation (LTP) [53]–[56], and consequently for these experiments, we modeled only the potentiation side of unsupervised burst-STDP. As we will show in Section, this is sufficient to learn invariant features of the environment. This bias towards potentiation, which could possibly destabilize network activity, is counterbalanced by a homeostatic mechanism (see Section). In our current implementation of our learning algorithm, we have placed an upper bound of 1.5 and a lower bound of 0 on the synaptic strength that occurs during training periods. While placing an upper and lower bound on synaptic strength somewhat opposes the results found in [46], maintaining realistic synaptic weights that converge on simple binary values (and thus would be more easily realized in silicon neuromorphic hardware) is a major goal of our neural system architecture. Through this learning mechanism the repeated presentation of a stimulus results in the selective strengthening of those connections relaying persistent inputs to output neurons.

#### Value Dependent Learning

Reinforcement learning is implemented by simulating the role of neuromodulators in signaling reward and punishment [13], [57]. Neuromodulators such as dopamine and noradrenaline are responsible for much of the reinforcement learning that happens in biological systems. In our model, a supervisory system outside of the actual neural network is used to evaluate the response of the network to a stimulus during training and reward or punish the appropriate synaptic connections contributing to the response. A correct reward is followed by multiplying the previously computed burst-STDP plastic change (Equation 3) by a positive constant, and a negative constant is employed for wrong responses. Moreover, the constants we employed for reward and punishments varied during the course of training, according to a process of simulated annealing [58], similarly to what has been shown to take place during development, i.e. a progressive decrease of synaptic plasticity [59]. This can be summarized by a modulation of the learning rate 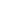:
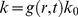
(4)


Here 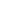 is the predetermined value of the learning rate (see the previous section) and 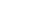 is the modulation performed by value-gating, which depends on both rewards 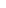 and time 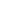. In summary, if the networks responds correctly to a stimulus, 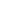 will affect the learning rate 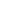 by making it positive, otherwise negative. Furthermore, early in the learning process learning rates will have higher absolute values to promote plastic changes.

In the initial phase of training, the reward constant is set to 0.5 and the punishment constant is set to −0.1. Thus, potentiation is always stronger than depression and, at the same time, connectivity can easily be changed from its initial condition. In the course of training, we set the punishment constant to 0 and gradually reduced the reward constant to 0.1. By observing the training of our network during experiments, we verified that including punishment helped perturb the synapses out of their initial condition quickly. However, as synaptic connections became more refined, training with positive reward alone is enough to achieve robust learning in the supervised regions of our model.

#### Renormalization

Homeostatic renormalization of synaptic strength has been hypothesized to take place during NREM sleep [15], [16] and be responsible for counterbalancing the predominance of potentiation occurring during waking time. A growing body of literature supports this hypothesis, showing that average synaptic strength, as well as its correlates in terms of neural activity, increase during waking and decrease during sleep [54], [55], [60]–[62] in a self-regulatory fashion [63]. One of the hypothesized consequences of synaptic renormalization is its contribution to memory consolidation, which we have recently shown in a large scale model of the thalamocortical system [50]. As we have described, burst-STDP works by mainly potentiating synapses, either for the repeated presentation of a stimulus (unsupervised learning) or because neuromodulators reward the correct response of the network. Thus a renormalization procedure is necessary to avoid a saturation of connection strengths.

Recently, we have proposed that renormalization may be implemented by a combination of both global and local mechanisms [50]. Previous *in vivo* works showed that changes in the levels of neuromodulators between waking and sleep may drive plastic processes towards either potentiation or depression [64]. This global mechanisms would favor potentiation during waking and depression during NREM sleep. At a single synapse (local level), instead, the weight-dependent properties of STDP [46] might play a fundamental role in memory consolidation. It has in fact been shown that the stronger a synapse is, the the smaller its strength changes will be following plastic events, in relative terms [46]. Thus stronger synapses will tend to remain fairly constant compared to weak ones. It can therefore be seen how a renormalization process following learning will play a role in memory consolidation, by preserving strong, trained synapses and pruning weak ones.

The renormalization we implemented here is a simplified version of the mechanisms we just described and works by linearly rescaling all incoming synaptic connections of a neuron so that the highest synaptic connection is set to 1. The 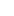 connection 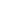 is therefore changed according to:
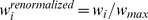
(5)


All incoming connections for a certain layer are renormalized simultaneously after a predetermined number of simulation steps. This is similar to having a period of waking (potentiation) followed by an offline sleep session (renormalization). We found the best delay between subsequent sleep sessions varied from layer to layer (from 500 to 2500 time steps on average). Intuitively, the optimal delay between such sleep sessions is related to the learning rate. However, it is also related to the particular learning tasks, as strong salient inputs drive the degree to which burst-STDP modifies the synapses. Renormalization promotes memory consolidation by setting the strongest connections to a value of 1 and progressively weakening unused ones, until they become negligible.

This homeostatic normalization process automatically binarizes synapses. This can be understood by considering one neuron and all its inputs. At the beginning, all synaptic strengths are uniformly distributed in the 0–1 range. Just before the first renormalization occurs, some connections will have been potentiated, and others depressed. After renormalization, the strongest connection will be set to 1, and all others will be weaker. Thus, during the next training period, the strongest synapse will be the one with the highest likelihood of causing spikes in the post-synaptic neuron, and therefore the highest likelihood of being strengthened. At every renormalization, the difference between stronger and weaker synapses will increase, until some connections will be fixed to a value of 1, and all others will be virtually 0. While in our experiments we notice that it is typically the case that synapses have converged, we utilize a simple threshold function to guarantee that synapses are set to binary values before testing. The major benefit to this binarization is that such simple synaptic weights are much more easily realized in actual hardware, where single synapses could be easily modelled as open (0) or closed (1) gates. While some level of resolution may be lost using a neural network with only binary synapses, we note that the results presented in this paper were collected after learning and binarization of the synapses, and thus show robust performance is still possible with such simplifications.

### Neural System Architecture

**Figure 2:**
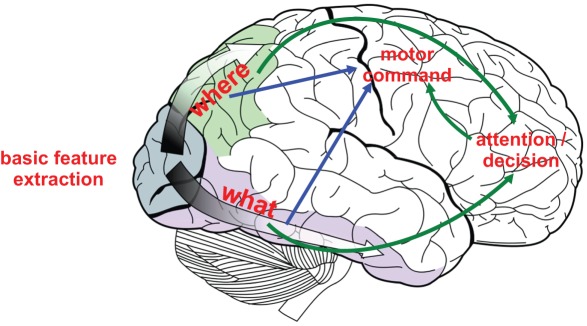
Basic architecture of the visuo-motor system. Primary visual areas perform basic feature extraction. The dorsal and ventral stream analyze the extracted information in terms of, respectively “where” and “what” content. A motor command is then elaborated in motor areas, with the contribution of prefrontal regions, devoted to tasks of higher complexity.

**Figure 3:**
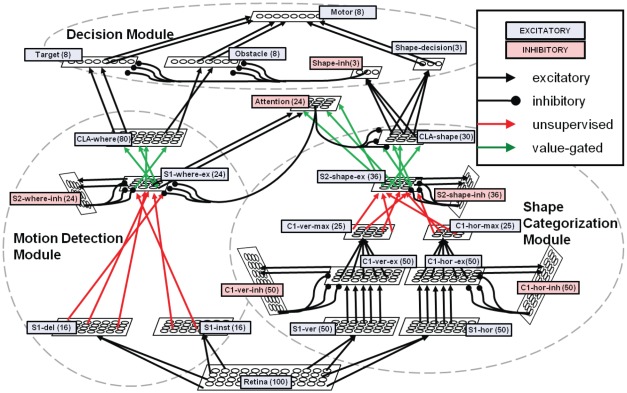
The architecture of the biologically inspired neural network. Each layer is depicted as a grid of cells (dimensions do not correspond to actual layer sizes) and all connections are showed. Parallel or converging connections represent topographic connectivity without or with dimensionality reduction; overlapping connections represent random connectivity. Subsystems consisting of multiple neural layers are grouped with dashed lines; four modules have been designed: a shape categorization module, a motion detection module, an attention module and a decision module. All hard-wired synaptic connections are black, and STDP learned connections are colored (red: unsupervised learning; green: supervised learning).

In the subsequent section, we describe in detail the hierarchical neural network architecture. This system is inspired by the anatomical and functional connectivity of several different brain areas. Figure 2 depicts the interaction among areas of the visual cortex for motion and feature processing, the prefrontal regions where decisions are made, and the motor area of the brain which interacts with the outside environment. Likewise, our biologically inspired neural network is composed of similar modules: the shape, motion, attention, and decision subsystems. The overall system architecture is depicted in Figure 3, and Table 2 describes in detail each of the neural layers used in the entire neural network. As motivated earlier, the choices we made in neuron and synapse models are highly motivated by neuromorphic hardwares. For this reason as well, we constrained our entire neural network to be composed of less than 1000 neurons, with the goal of demonstrating a minimal, yet scalable, network architecture.

**Table 2:** Each group of neurons in the network described in detail.

Module	Layer	Neuron Type	Size	Target Layers	Topographic RF Size	Train Only	T	L	S+	S–
**Retina**	Retina	Ex	10×10 (100)	All S1 cells	1×1	N	N/A	N/A	N/A	N/A
**Shape**	S1-ver	Ex	10×5 (50)	C1-ver-ex	1×2	N	31	16	16	0
**Shape**	S1-hor	Ex	5×10 (50)	C1-hor-ex	2×1	N	31	16	16	0
**Shape**	C1-ver-ex	Ex	10×5 (50)	C1-ver-inh, C1-ver-max	1×1	N	4	2	8	64
**Shape**	C1-hor-ex	Ex	5×10 (50)	C1-hor-inh, C1-hor-max	1×1	N	4	2	8	64
**Shape**	C1-ver-inh	Inh	10×5 (50)	C1-ver-ex	1×1	N	4	2	32	0
**Shape**	C1-hor-inh	Inh	5×10 (50)	C1-hor-ex	1×1	N	4	2	32	0
**Shape**	C1-ver-max	Ex	5×5 (25)	S2-shape-ex	2×1	N	1	2	32	0
**Shape**	C1-hor-max	Ex	5×5 (25)	S2-shape-ex	1×2	N	16	2	32	0
**Shape**	S2-shape-ex	Ex	6×6 (36)	CLA-shape, Attention, S2-shape-inh	N/A	N	100	64	32	255
**Shape**	S2-shape-inh	Inh	6×6 (36)	S2-shape-ex	1×1	Y	2	32	32	0
**Shape**	CLA-shape	Ex	3×10 (30)	Shape-inh, Shape-decision	N/A	N	16	2	32	128
**Motion**	S1-inst	Ex	4×4 (16)	S2-where-ex	4×4	N	27	26	4	0
**Motion**	S1-del	Ex	4×4 (16)	S2-where-ex	4×4	N	27	26	4	0
**Motion**	S2-where-ex	Ex	4×6 (24)	CLA-where, Atention, S2-where-inh	N/A	N	100	64	64	192
**Motion**	S2-where-inh	Inh	4×6 (24)	S2-where-ex	1×1	Y	16	2	32	0
**Motion**	CLA-where	Ex	8×10 (80)	Target, Obstacle	N/A	N	32	16	64	0
**Attention**	Attention	Inh	4×6 (24)	S2-where, CLA-shape, Attention	N/A	N	5	16	16	128
**Decision**	Target	Ex	1×8 (8)	Motor	1×10	N	21	5	1	80
**Decision**	Obstacle	Ex	1×8 (8)	Motor	1×10	N	21	5	1	80
**Decision**	Shape-inh	Inh	1×3 (3)	Target, Obstacle	1×10	N	4	4	1	0
**Decision**	Shape-decision	Ex	1×3 (3)	Motor	1×10	N	13	3	1	0
**Decision**	Motor	Ex	1×8 (8)	N/A	N/A	N	8	7	4	0

As can be seen, the entire network is built around a modest set of parameters for the odeled neurons. Each column describes the properties of neuronal groups. Module: the architecture sub-system to which the group pertains. Layer: the neuronal layer in which the group is incorporated. Neuron type: either excitatory (Ex) or inhibitory (Inh). Size: the shape of the rectangular layer (between parentheses the total number of neurons). Target layers: the projecting layers for the group. Topographic RF size: the receptive field of each neuron (N/A if not applicable). Train only: whether the group is active only during training (Y) or not (N). T: firing threshold. L: leakiness factor. S+: excitatory weighting value. S-: inhibitory weighting value.

#### Model Neurons

Models of cortical neurons and biological neural networks can vary extensively in terms of their biological plausibility and computational efficiency [40]. These models can span from the simplest implementation of memoryless perceptron based neural networks, to the highly complicated Hodgkin-Huxley model which emulates details such as ion channels and neural conductances in biological systems. The neurons we have simulated for this paper are classified as leaky integrate-and-fire (LIF) neurons and are modeled according to the previously outlined hardware constraints. We simulate both excitatory and inhibitory neurons with a number of configurable parameters. Each neuron has a local membrane potential (*M*), leakiness factor (*L*), and firing threshold (*T*). The synapses we model are a combination of simple hard-wired connections, purely unsupervised burst-STDP synapses, and supervised value gated burst-STDP synapses. While synaptic strengths may vary during training epochs, the homeostatic renormalization rule we employ guarantees that synapses will eventually converge onto binary solutions. Each neuron also has a multiplicative synaptic weighting value which determines how much an excitatory (*S+*) or inhibitory (*S−*) synaptic input will affect the membrane potential. At each simulation cycle, *M* is updated according to the Equation 6 below, where *N* is the total number of excitatory synapses and *O* is the number of inhibitory synapses of a given neuron. If the membrane potential is greater than the firing threshold *T*, the neuron fires and *M* resets to zero. *X* and *Y* are respectively the excitatory and inhibitory presynaptic inputs to the model neuron.
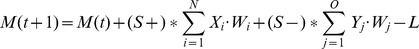
(6)


In many LIF neuron models, the membrane leak factor is proportional to the current membrane potential [40]. However, as motivated earlier, a digital implementation of the LIF may utilize a linear leak factor with a lower bounded membrane potential to meet power, area, and complexity constraints. Likewise, our modeled neuron uses a constant, linear leak factor *L*. In order to avoid negative values for *M*, the membrane potential utilizes a lower bound of 0 V.

#### Shape Categorization Module

The shape categorization module provides the translation-invariant recognition of an object in the environment. Our shape categorization module is similar in nature to Poggio's HMAX [20] as well as Masquelier's STDP implementation of HMAX [24]. Like these visual cortex models, the shape categorization stream alternates simple cells (S) which elicit a spiking response for their preferred input and complex cells (C) which provide translation invariance by using a max pooling operation over a population of simple cells. The overall architecture of the shape module creates a four layer hierarchy (S1-C1-S2-CLA), with the top level of the hierarchy being a classifier. Our current implementation of this architecture is a simple one, employing only a single processing scale and two preferred edge-orientations (vertical and horizontal lines) at the lowest level. While this work focuses on the abilities of LIF neurons using the burst-STDP learning in a very basic hierarchical architecture, we have performed preliminary testing to ensure our design can easily be expanded to incorporate more preferred edge-orientations, processing scales, and neuron groups.

As in the visual area V1, the first layer of our hierarchy (S1) acts as a simple edge detector [18]. The S1 cells are tuned to respond maximally to a particular edge of a certain orientation. A key design feature of our network is that the LIF neurons used are able to maintain a membrane potential between simulation time steps, as opposed to resetting the membrane potential for each evaluation of the network. This leaky membrane potential, as opposed to a memoryless membrane potential, allows the neuron best tuned to a particular edge to respond first, even if that edge is not a perfect match (as in the case of a noisy environment, or slight variation of the same feature).

At each time step, the outputs from the S1 neurons propagate to the C1 cells, which is responsible for propagating the spikes of the maximally responsive S1 cells to the higher levels of the system. In the shape categorization module, the C1 area for a particular edge orientation is composed of three groups of neurons to properly implement a max pooling function [65], [66]. To illustrate the design, we will consider the C1 area preferring horizontal edges in Figure 3. In this area, C1-hor-ex are excitatory neurons strongly connected to the corresponding S1 neurons directly beneath them. The C1-hor-ex neurons are connected as the presynaptic inputs to a set of inhibitory neurons known as C1-hor-inh, which in turn project their inhibitory outputs back to a localized area in the C1-hor-ex neurons. This localized inhibitory behavior allows the C1 layer to filter out noise as well as propagate only the strongest edge-detection responses for a given receptive field (or neighborhood of neurons). Finally, C1-hor-ex cells also project to the excitatory C1-hor-max neurons, which converge the responses over a neighborhood of C1-hor-ex neurons. The lower levels of the shape categorization module (S1–C1) are hard-wired neural connections which separately observe the environment for each preferred orientation. Each of these layers is a retinotopically organized 2D grid of neurons, with each neuron's location corresponding to the region of the visual environment where it receives its receptive field inputs (whether directly from the retina or through other retinotopically organized neurons).

The S2 layer combines the responses of C1 neurons from the different orientations of preference using the unsupervised burst-STDP learning rule. Similarly to the C1 neuron layer, the S2 is composed of multiple populations of neurons. First, the connections between the C1-max neurons and S2-shape-ex neurons are initialized to random weights. The S2-shape-inh neurons are activated by the firing of a S2-shape-ex cell, which have reciprocal connections back to the S2-shape-ex region to inhibit neighboring cells. In this way, the S2-shape-ex cell that first responds will be reinforced with the STDP learning rule, while its neighbors will not since the S2-shape-inh cell has inhibited them from firing. This competition creates a weakly-enforced winner-takes-all (WTA) method to encourage neurons to learn different objects. We do not consider this a strictly-enforced WTA, since nothing prevents two S2-shape-ex cells from starting with the same random weight connections, activating, and strengthening their synapses at the same time–though this behavior has been observed rarely in our experiments. Because of the initial random connectivity of this neural layer, the S2-shape-ex neurons' receptive fields are not retinotopically organized as in the lower level neural layers. While this connectivity means that a certain level of detail is lost to the upper levels of the shape categorization module, it does not hinder the performance of the system given the simplicity of the current retina implementation and the scale of neural system we are interested in modeling. Future extensions to our model may justify the use of maintaining a topographical organization for the higher neural modules, and we have performed some initial experiments which utilize topographical receptive fields in the S2 neural layer.

Finally, the uppermost layer of the shape categorization module is the classifier (CLA-shape), which learns to invariantly recognize a particular object anywhere in the visual environment, similar to the IT region of the visual cortex. While using a single neuron per shape or object classification worked in our initial experiments (i.e., one neuron learned to fire invariantly for the presentation of the letter ‘T’ anywhere on the retina), we found that learning rates were significantly improved using a population, or pool, of neurons per learning category. Given that the network has been trained long enough, various S2-shape-ex neurons will consistently fire in response to an object stimulus as it moves through the environment. Each category pool of neurons is initialized with random connectivity to the S2-shape-ex neuronal layer, and the synapses are strengthened using the value gated burst-STDP described earlier. The main advantage of using a neuron pool is that the response of each neuron in the pool will depend on its initial connectivity, which in turn will elicit rewards or punishment. The more neurons that are activated, the more the reward system induces the value gated burst-STDP, which ultimately drives the CLA-shape layer to learn the appropriate categorizations of the S2-shape-ex layer neurons. We note again that other bio-inspired models for visual categorization have been developed (see for example [21], [67], [68]). However, the scope of our work was not to develop a model able to outperform existing ones, but rather to show the capabilities of a minimal, *in silico*, fully neuromorphic approach, and HMAX provided a good, widespread framework.

#### Motion Detection Module

The architecture of the motion detection module draws inspiration from previously published works in the field, such as [27], [28], which are based on an HMAX-like architecture. This approach, far from being the only bio-inspired model present in the literature, has the advantage of allowing us to implement an architecture similar to the shape categorization model, thus increasing the modularity of the whole network. Therefore, the architecture of the motion detection module is organized hierarchically like the shape categorization module and features both hard-wired and plastic/learned synapses. First, a hard-wired feature detection step exhibits firing for a preferred stimulus location. Next, unsupervised learning associates the detections across multiple of these hard-wired feature detectors. Finally, supervised learning via value gated burst-STDP categorizes these associations.

Motion is first divided into basic steps–or primitives–which are then combined into higher level paths. Our implementation–although simplified to account only for few straight-line paths–is however based only on spiking neurons and is therefore a starting point for designing fully neuromorphic motion detection models.

The feature detection step is made up of 2D retinotopically organized neural layers, S1-inst and S1-del. Both receive inputs from the retina and have the same receptive fields, but the connections between the retina and S1-del introduce a delay that allows the network to detect the motion of a shape. Thus, if a shape can only move every 10 time steps, the optimal delay will correspond to 10 time steps as well. The receptive field of each neuron covers a 4×4 area of the retina, and each neighboring neuron in the S1-inst and S1-del layer overlaps its receptive field by two pixels with its neighbor in both the horizontal and vertical directions. These neurons–which are memoryless in the sense that they are highly leaky and do not maintain a membrane potential between subsequent time steps–have a threshold which allows them to fire whenever any of the input stimulus shapes is present in their receptive field. Thus, these cells show no preference for a particular object, but simply fire when an object is present at all.

Next, unsupervised learning forms connections between the S1 and S2 layers in the motion detection module. The S2 area in this module consists of two neural layers, S2-where-ex and S2-where-inh, both composed of 24 cells. Each cell in S2-where-ex is connected by random weights to all units in both S1-inst and S1-del. Cells in S2-where-inh receive inputs from a single corresponding cell in S2-where-ex, and reciprocal output connections inhibit all other S2-where-ex neurons. In the training phase, only one shape is present at any given time on the retina (without noise); thus only one cell in S1-inst and S1-del are active simultaneously. The combination of the S2-where-ex and S2-where-inh again create a weakly enforced WTA network. Since connections from the S1 layers to S2-where-ex are initialized with random synaptic weights, it is likely that a single S2-where-ex neuron will build up membrane potential, fire, inhibit its neighbors through the S2-where-inh cells, and update its synaptic connections through burst-STDP. Because the initial connectivity is random, nothing strictly enforces that only a single cell wins every time, but in practice each S2-where-ex neuron typically learns a unique combination of S1-inst and S1-del cells. At the end of training, cells in S2-where-ex will be specialized for a particular combination of S1-inst and S1-del spikes, i.e. for a particular localized direction of motion.

Finally, the value gated step works by associating several S2-where-ex units to a corresponding direction and starting point combination. Each direction/starting point combination is represented by a pool of neurons in layer CLA-where. Here we modeled eight different direction/starting point combinations (two directions of motion are possible at each starting point in each of the four corners of the retina) and each pool contains ten neurons. Again, eight neurons (one per category of motion) could achieve this task, but we found using pools of neurons improved learning rates for the system. Whenever activity integrated over time in a pool is greater than that of the other pools, the external reward system is activated, and a subset of connections is potentiated or depressed, depending on whether it is the correct or wrong pool that is firing. After extensive training, each pool groups several S2-where-ex cells (each of which has learned a small, localized preferred direction of motion) through its synaptic connections. As such, the same pool of neurons in the CLA-where neural area will fire consistently as an object moves all the way across the retina.

This architecture is a simple yet effective model of cortical motion detection systems. S2-where cells perform the most basic motion detection step, by comparing the position of features in subsequent steps, each along one predefined direction. Thus they basically replicate (in a very simple manner) the role of the medio-temporal cortex (MT). CLA-where cells, instead, represent higher areas, such as parietal ones, where this information is integrated over larger receptive fields.

#### Attention Module

Because of the vast amount of raw data the retina provides to the visual cortex, it is useful to have a mechanism to discriminate important features and objects from other distractors. The attention module accomplishes this important task by providing top-down signaling to the lower level visual processing areas through feedback connections to place emphasis on important features and filter out distractors. In our network, the visual attention neurons perform this task when multiple objects are present; they provide focus on the objects in the visual receptive field determined to be the most important through feedback connections, while silencing the neurons firing for distractor objects.

As can be seen in Figure 3, the attention module receives excitatory input from both the shape categorization and motion detection modules. While the attention module receives topological hard-wired connections from the motion detection module, the synapses from the shape module are initialized to random weights and strengthened through value gated burst-STDP. During training, the value gated burst-STDP only modifies the plasticity of these connections when the target object is present in the retina. In this way, the attention module learns associations between the directions of motion, as well as the neurons in the S2-shape-ex layer that fire for a particular presentation of a target object. During learning, attention neurons inhibit their neighbors to promote learning of unique shape and motion pairs. In this way, a single attention neuron should fire whenever a target object is present in the retina.

After learning has completed, the attention module sends top-down inhibitory connections to both the motion detection and the shape categorization modules. In the shape categorization module, the attention module inhibits all neurons in the CLA-shape layer except for those firing for the target object. Since the connections from the S1-where-ex layer are topologically organized, the firing of an attention neuron indicates both that the target object is present, as well as its local range of motion. In turn, the attention module projects reciprocal connections back to the S1-where-ex layer, inhibiting any motion detection that is not associated with the target object.

In this way, attentional modulation is first triggered bottom-up by the recognition of a target object. Afterward, attention provides top-down inhibition to filter out distractor objects in the CLA-shape layer as well as distractor motion in the S1-where-ex layer. This inhibition also provides some noise filtering for both neural layers. This method of global inhibition was first proposed by Fukushima [69], though more localized attentional modulation systems have been proposed [70] and experimentally validated [71].

Other attention models have been proposed in the context of object recognition, with most recent works focusing on saliency as a tool to extract relevant features. One example is bottom-up attention based on salience [72], [73], which has also been shown to work in conjunction with HMAX [74]. Interestingly, saliency has been applied either as a tool to extract the most relevant features from the environment [75], or as a way to focus further computational efforts on significant features only [76], [77]. A combined bottom-up and top-down approach has also been introduced not only to extract relevant features, but to modify eye position accordingly [78], thus implementing a feedback loop. Finally, attention has been proposed as a strategy to focus reinforcement learning on the most behaviorally relevant circuits [79]. In our network, instead, the attention module has been designed to play a more focused role in a simple yet effective manner. Thus, rather than focusing network activity on the most relevant features present in the visual field, the attention module has to select the most behaviorally relevant object among several ones that may appear simultaneously. It must also be pointed out that, although the attention module has been designed specifically for the task of discriminating between two classes of moving objects, it could easily be adapted to more complex cases, the only limiting factor being the number of available artificial neurons.

#### Decision Module

The decision module is responsible for determining the motor output reaction to the state of the visual environment. This decision module helps the network cope with the presence of noise in the input environment. In particular, it evaluates whether the classifications performed by the shape and motion detection modules are consistent over time or just sporadic detections (and thus likely to be erroneous detections caused by noise).

**Figure 4:**
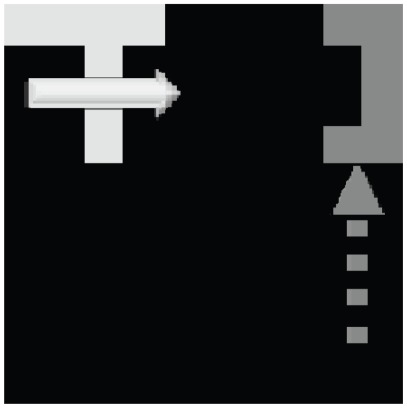
The 10×10 pixel simulation environment, as seen by the retina. The object (here a white “T”) appears in the upper left corner and moves along the top edge of the visual field. After learning, the decision module motor output moves the catcher (visualized as the dark grey object) from its previous location to a location and orientation where it will catch the object.

There are several neural groups that make up the decision module and are ultimately responsible for making the motor output decision. The Target and Obstacle neuron groups are hard-wired to the CLA-where neuron layer, competing to activate the motor output given the particular motion detection that has been classified. In the current implementation, the “high level” motor output is the decision to avoid an obstacle or approach a target, while the lower level motor outputs determine the precise motor outputs required to achieve this decision. Biologically, we consider how a mammal may make the high level decision to get some food or avoid a predator, while lower level motor outputs actually orchestrate the motion and minute actions. The Target neuron group attempts to activate the motor neuron pool which moves the “catcher” (see Figure 4) to the destination of the target object, while the Obstacle neuron group moves it to the furthest corner. The Shape-inh is a layer of inhibitory neurons which are activated by the shape categorization module CLA-shape neurons (i.e. they are easily activated by having a very low firing threshold), which in turn inhibit either the Target or Obstacle neuron layers depending on the current shape categorization. Finally, the Shape-decision neural layer is also activated by the CLA-shape neurons, though they require consistent activations of the same shape over multiple time steps before activation (i.e. they have a high firing threshold, requiring many activation inputs), thus creating a robust classification even in a noisy environment. The motor neurons activate once simultaneous firing occurs between the Shape-decision and either the Target or Obstacle neurons.

## Results

We evaluated the performance of the network using several different recognition tasks. For these tasks, we trained the network in a noiseless environment on a single object at a time using a 10×10 pixel visual stimulus environment. The choice for this simple visual environment again was motivated chiefly by our desire to show a minimal multi-modal network architecture of less than 1000 neurons. Given this strict constraint, we were able to develop a network in which 100 neurons (10% of all cells) were employed as a noisy retina, and the rest of the network was still able to discriminate two orientations, three different objects – also appearing simultaneously in the visual field, eight possible trajectories, and associate a distinct behavior to each object. Although the environment was necessarily simple, we are nevertheless able to show the potential of our architecture. In the following experiments, we show the network's abilities to classify multiple simple objects, track motion in a noisy environment, and make correct motor outputs for multiple moving stimuli in a noisy environment. We also demonstrate the necessity for the top-down modulation provided by the attention module.

### Learning Multiple Categories

**Figure 5:**
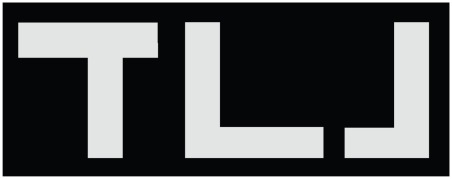
The trained stimulus of the neural network. “T” is a Target object, the catcher must be positioned in front of it; “L” is an avoidance object, the catcher must be placed in the position opposite to it; “J” is a distractor and should not elicit a motor response.

**Figure 6:**
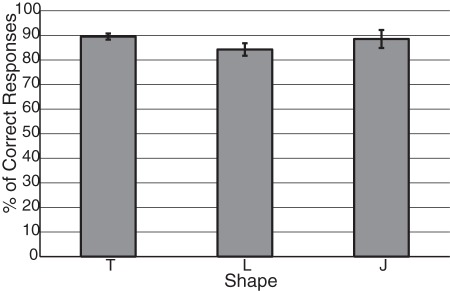
Performance of the shape categorization module. This graph shows the percentage of correct responses to the presentation of the three letters in each possible position in the visual field (mean values 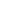 standard errors, 4 different random initial conditions for connection strengths). Each initialized network was trained for a single epoch. A correct response is given around 80% of times or more for all letters.

Our first experiment tested the network's ability to learn translation invariant representations of multiple objects as they move through the visual field. In the training phase of the experiment, we presented a single object at a time, which appeared in one of the four corners of the retina, moved in the lateral or vertical direction, and finally moved out of the receptive field of the retina (see Figure 4) The corner where the object initially appeared, as well as its direction of motion, were chosen at random. This experiment used three different objects, the letters: T, L, and J, in both the training and testing phases (see Figure 5). Figure 6 shows the average performance of the shape classification module's response after a single training session. Here we tested four different random initializations of connection strengths with the same training set, in order to verify the robustness of our network in categorizing the shown stimuli. Testing consisted of 100 presentations of each letter, with the starting corner and direction of motion chosen at random. We see that the average correct recognition of each of the letters was between 80 and 92% after a single training epoch. The variance of recognition across trials was predominately a result of variable random initialization of the plastic synapses. For all subsequent experiments where the degree of noise on the retina is varied, the network was trained for multiple epochs to ensure robustness.

**Figure 7:**
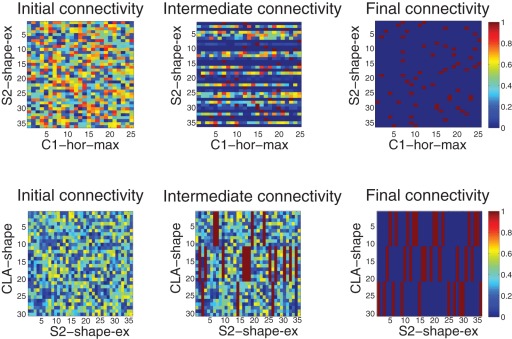
Changes in connectivity as a consequence of training and burst-STDP with renormalization in the shape categorization module. Top: unsupervised learning, connections from layer C1-hor-max to layer S2-shape-ex. Bottom: value-gated learning, connections from S2-shape-ex to CLA-shape. In each subfigure, the X-axis is the presynaptic layer, and the Y-axis is the postsynaptic layer.

Figure 7 shows how the connectivity differed before, during, and after training for the shape categorization module. In the top of the figure, we examine the synaptic connections from the C1-hor-max neural layer to the S2-shape-ex layer. Initially, the connectivity is randomized (top left of Figure 7), with no designed bias. However, during training we see that various plastic synapses have strengthened, and homeostatic renormalization has ensured that the maximum synaptic strength is 1. After training, we see all of the S2-shape-ex neurons have formed just a few strong synapses with the C1-hor-max neurons. These S2-shape-ex neurons have also formed strong synaptic connections with neurons in the C1-ver-max layer, and as a result an S2-shape-ex neuron will fire for a single shape at a specific position in the retina. Above the S2-shape-ex layer, the CLA-shape classifies the various S2-shape-ex cells into three categories corresponding to the three learned shapes (bottom of Figure 7). Again, initial random connectivity does not show a designed bias for a particular learned set of synapses, but we see the final connectivity classifies nearly all S2-shape-ex neurons into one of three pools (bottom right). Thus each S2-shape-ex cell will lead to the activation of one of the three pools in CLA-what layer, which will classify shapes independently from their position on the retina (position invariance). It can also be seen from this figure that the homeostatic renormalization has converged the synaptic weights, initialized as random, to binary values, since each synapse is a an on-synapse (red pixel) or an off-synapse (blue pixel).

### Motion Detection in a Noisy Environment.

**Figure 8:**
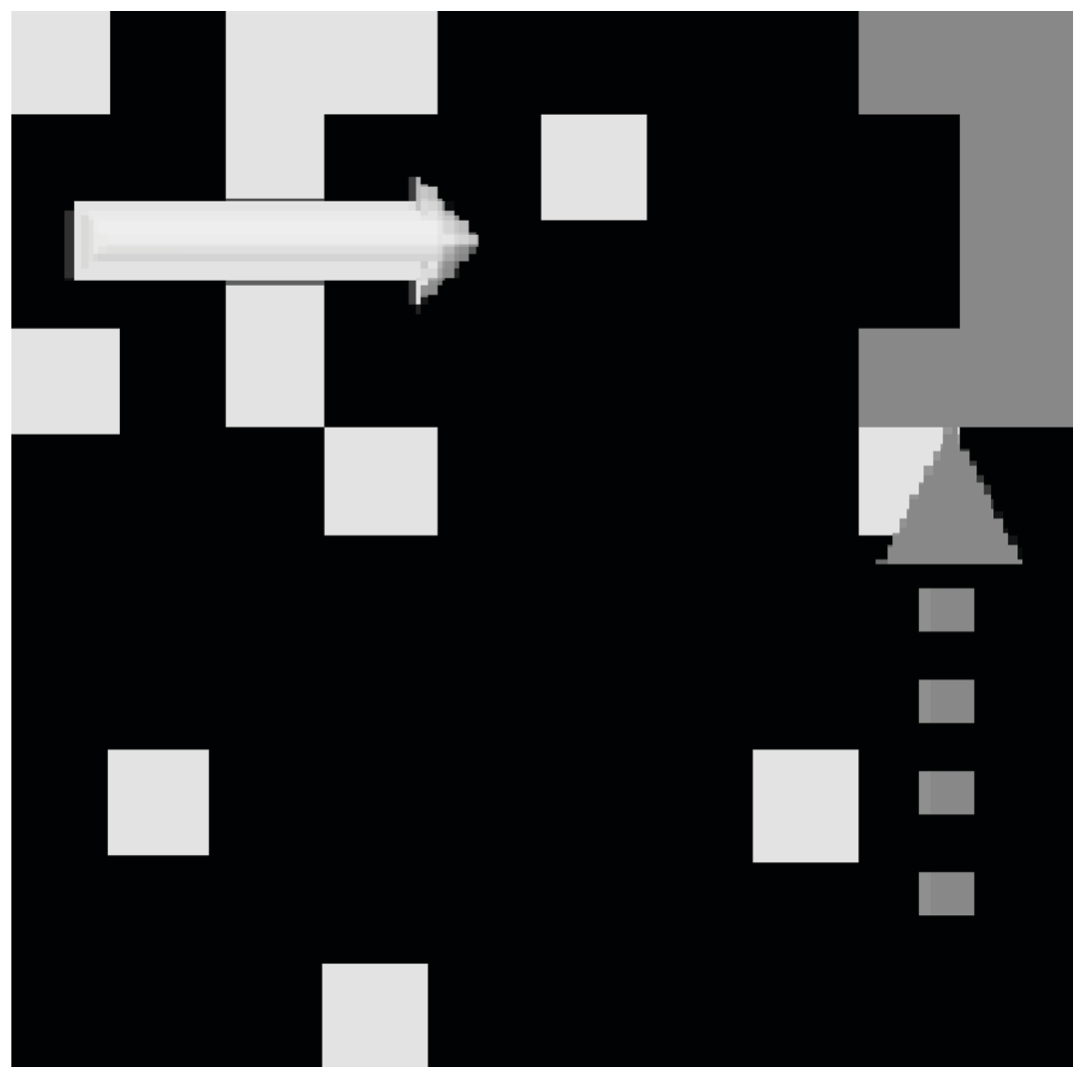
Visual environment with 8% noise injection. The object (here a white “T”) appears in the upper left corner and moves along the top edge of the visual field. Noise has been modeled as an 8% probability of changing the value of any pixel (from 0 to 1 or vice versa). The catcher is correctly moved to the top right corner, facing the arrival of the target.

Our second experiment tested the motion detection ability of the network in a noisy environment (as in the example of Figure 8). Again, in the training phase of the experiment, the retina was presented with a single object at a time, appearing in one of the four corners. The object then moved in a lateral or vertical direction until it moved outside of the receptive field of the retina. Since the retina is organized as a simple square, the amount of time an object is present in the retina is the same from trial to trial. The same three simple letters (T, L, and J) were chosen randomly with equal likelihood. For each trial, the response of the network was interpreted as follows. If no motor response was recorded between the time a new object appeared and when it had moved out of the retina's receptive field, the response was considered a “No Decision” However, if a motor response was recorded during this time, the last response was considered to be the final decision. For example, if initially the network responds with a correct decision, but then changes to an incorrect decision before the object dissappears, the network's response is considered “Incorrect”.

**Figure 9:**
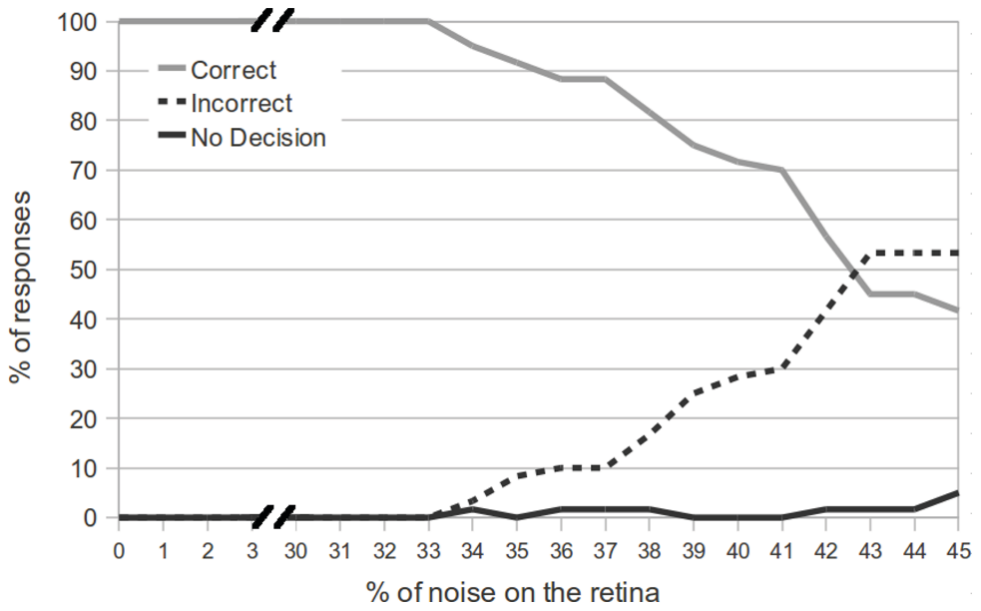
Performance of the network at motion detection task. The graph shows the performance of the network for the motion detection task as a function of the % of noise on the retina. A “Correct” response means the last motor decision of the network matched the direction of motion, and an “Incorrect” response means the last motor decision of the network did not match the direction of motion for the stimulus. Finally, “No Decision” indicates that no motor response was recorded. The network shows 100% accuracy until the noise on the retina is above 33%.

Figure 9 shows the results of this experiment, as the level of noise is increased up to 45%. We see from the figure that the network is able to correctly classify (with 100% accuracy) the direction of motion, even with 33% noise on the retina. As the level of noise continues to increase, the network begins to make incorrect decisions. For a noise level greater than 42%, an incorrect decision becomes more likely than a correct decision. However, overall the results show that the motion detection module is quite robust.

### Full Network Response in a Noisy Environment

We next evaluated the performance of the entire network in the presence of a noisy environment.

In biology, the inputs to the visual cortex always exhibit some level of noise, yet somehow is able to make sense of its surroundings. Since the cells modeled in our neural network are LIF, the cell membranes will still potentiate in response to noisy inputs and maintain a memory across multiple time steps, so long as the noise is within a reasonable limit. As a result, the network has an inherent resilience to filter out much noise on its own, as even noisy inputs will eventually cause the cell tuned for a particular edge, feature, or object to fire. Additionally, the decision module makes the network more robust by determining if classifications performed by the shape and motion detection modules are consistent over a reasonable time interval.

We varied the total amount of noise in the visual receptive field for a given simulation cycle from 0 to 25%. That is, in the 10×10 pixel environment, if there is 8% noise injection, on average 8 pixels will be flipped at any given time (see Figure 8). Again, the object, the starting position, and direction of motion are chosen at random. A response is considered correct if the correct motor output neuron fires before the object has moved out of the visual environment. The response is considered incorrect if the motor output is to the wrong location for target and avoidance objects. For these practical purposes, tests were conducted using only the T-shape (target) and L-shape (avoidance object). Finally, all other responses are categorized as non-decisions, in which no motor output was chosen at all.

**Figure 10:**
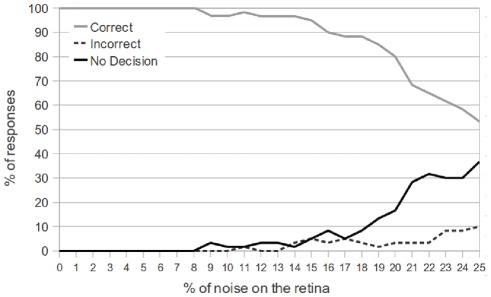
Performance of the network with single object presentation and a varying level of noise. The graph shows the performance of the network when a single object is presented, as a function of the % of noise on the retina. All letters, positions and directions of motion were tested. The network responds correctly up to 8% noise on the retina. When the noise on the retina is above 18%, the number of non-decisions begins to rise rapidly. However, even for 25% noise on the retina, the number of incorrect decisions is less than 10%.

In Figure 10, we see the results of the network performance as noise is increased. The testing phase (for each percent of noise injection) consisted of 100 object presentations, and the target object and avoidance object were chosen with equal probability.

We see that the network is able to always give a correct response for up to 8% noise injection on the retina. As the degree of noise is further increased, the number of non-decisions begins to rise, and the number of correct decisions falls. The network responds correctly 80% of the time with 20% noise injection, and 54% of the time with 25% noise injection. However, as a result of the decision module, we see that the number of incorrect decisions is consistently low, only reaching 10% when 25% of the retina is exhibiting noise.

### Multiple Moving Objects

Next, we tested the response of the network in an environment where multiple objects could appear in the presence of noise. The network was trained over multiple learning epochs for this experiment, as well as those that follow. Extensive training for the network meant that most, if not all, synapses would converge on useful values, making the network more resilient to noise. In this experiment, the system was tested on a total of 100 presentations (for each percent of noise injection). On each presentation, 25% of the time a target object (T) appeared, 25% of the time the avoidance object (L) appeared, and 50% of the time both appeared. The starting position and direction of motion of all objects were chosen independently and randomly. During training, the attention module neurons were value gated to reward firing for presentations of the T–that is, to pay attention to the target object over the avoidance object. For this learning task, we trained the network to catch the target object, regardless of what other objects may be present.

**Figure 11:**
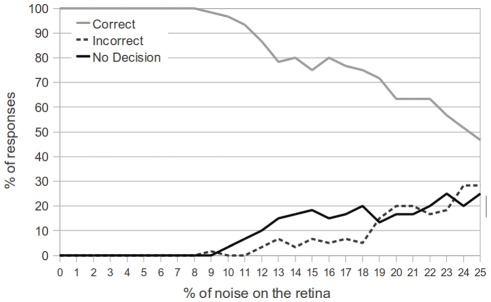
Performance of the network with two simultaneous objects presented and a varying level of noise. The results of the graph were obtained by testing the network on both single objects (50% of presentations) and two objects (50% of presentations) moving across a noisy retina. Again, the network is capable of 100% correct responses when retinal noise is below 8%. As the noise on the retina is increased, both the number of non-decisions and the number of incorrect responses increases.

Figure 11 details the performance of the network with a variable amount of noise injection. Here, the correct motor response is to catch the T if it is present and avoid the L if the target object T is not present. The results are quite comparable to those shown in Figure 10, as the network shows 100% correct responses even with 8% noise on the retina. Afterwards, the number of non-decisions begins to rise, similarly to the results shown in Figure 10. However, we also notice that the number of incorrect decisions is much higher when there is a high level of noise. When both a target object and an avoidance object are present at the same time, but the target object goes undetected because of noise, it is more often the case that the avoidance object will cause an incorrect decision. However, we see that the performance of the network degrades gracefully as the amount of noise is increased.

The scale and complexity of our network is minimal in comparison to many other learning models, and the stimulus space in which it operates is also very simple. Yet, we find its ability to robustly learn and perform tasks of object recognition and motion detection promising and foresee an expansion of the model in order to cope with more complex environments.

### Evaluating the Attention Module

**Figure 12:**
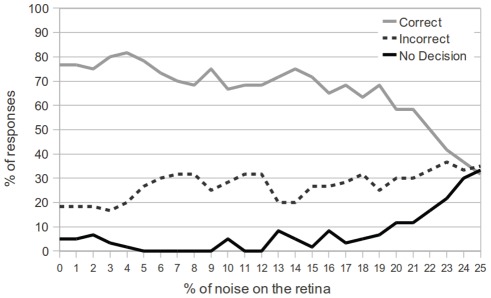
Performance of the network without attention module. The graph shows the same experiment as presented in Figure 11 after severing the outputs of the attention module. Even for low levels of noise in the retina, the network seldom achieves greater than 75% accuracy at the task. Furthermore, the number of incorrect responses is significantly higher (even for low noise levels), since the attention module no longer shows preference for the target object.

Finally, we demonstrate the importance of the attention module for the multiple moving objects task. The network tested in Figure 11 includes an attention module which utilizes top-down inhibition to mask both noise and distractor objects whenever a target object (i.e. the T object) is present. Figure 12 shows the exact same experimental setup with the same network, except the output connections from the attention module have been severed.

From the results, we see that even for very low levels of noise, the network seldom achieves greater than 75% correct responses. This is mostly due to the nature of the task. When both a target object and an avoidance object are present, the network should preferentially respond to the target object first. However, as we see here, without an attentional mechanism driving this preference, the network is much more likely to make mistakes, responding to the avoidance object instead. Comparing Figures 11 and 12, we clearly see the benefit of attentional modulation even for the simple tasks presented here.

## Discussion

In this paper we have presented a detailed network architecture of spiking neurons capable of both recognizing moving objects and determining the correct motor control output for a particular object. This hierarchical neural network was trained using a learning paradigm built around burst-STDP, value gated burst-STDP, and homeostatic renormalization. This biologically inspired learning algorithm takes into account the observed fact that spiking is expensive in terms of energy. Thus, bursts convey only the most certain information and promote faster learning rates than single-spike STDP. The combination of leaky integrate-and-fire neurons, a biologically inspired network architecture, and burst-STDP learning rule allowed our model to robustly perform its recognition and motor decision tasks, even in the presence of distracting objects and a noisy environment.

The main contribution of this work is a demonstration of how a simple neural model with a limited set of parameters and binary synapses can achieve robust performance at various tasks. It is clear how neural networks of this design are primary candidates for deployment on energy efficient neuromorphic hardware designs in silicon. As such, we have demonstrated that the simple, modular building blocks presented in this work can provide a proof of concept of the feasibility of our approach. Future work will deal with expanding the size and capabilities of the network, exploiting its modular properties in order to make it easy to test the network on simpler environments and then scale it with a reduced effort.

Previous works, for example [20], [21], [24], have shown that bio-inspired architectures can approach the performance of both state of the art image recognition computational techniques as well as human beings in some tasks. Recently, it has however been shown that biologically inspired models such as HMAX fail to outperform a V1-like model in the classification of reference image collections, thus raising doubts on their capabilities to be employed for uncontrolled natural images [22]. Nevertheless, we decided to employ HMAX as a starting point for our bio-inspired architecture for several reasons: HMAX is widespread not only in the artificial vision field, but in the whole neuroscientific community; its modular, scalable architecture is suitable for a hardware implementation; implementations for both object recognition and motion detection have been developed and allow to design a homogeneous, replicable architecture.

In this work, we attempt to follow a completely bio-inspired approached and therefore mimicked biological systems in terms of: 1) elementary units – we used actual artificial neurons with axons and dendritic trees (see the Hardware constraints sections for details on neuron design), rather than fitting neural activity with approximate functions; 2) architecture–although highly simplified, neuronal types and connectivity are based on actual brain organization, such as the existence of simple and complex cells and the hierarchical organization of cortical areas; 3) communication system–neurons use actual spikes for transferring information; 4) learning mechanisms–burst-STDP is directly derived from neural systems. The results show that even this simple bio-inspired system can successfully be employed to perform complex tasks such as shape categorization, motion anticipation, attention modulation, and decision making. Although the small size of our network architecture limited the visual stimulus environment to a 10×10 grid of binary pixels containing simple letter shaped objects, with less than 1000 neurons we were able to build simple models of multiple brain areas responsible for several important functions. We must here emphasize again that the goal of this work was to show the potential offered by a neuromorphic, *in silico* approach, and not to develop a novel computational model of the visual system. In fact, it would be quite difficult to compare our network with state of the art ones, given all the hardware-derived constraints we strictly adhered to.

The same hardware-driven constraints forced us to choose between designing a network able to cope with a single, complex problems, or able to perform multiple simpler tasks. We chose the latter strategy, since we felt it could show the versatility of a fully neuromorphic system. Although much work remains to be done, being able to perform different tasks is fundamental for applications in the field of robotics. As we will discuss in the following paragraphs, expansions to the present network are needed to increase its power to deal with more complex tasks and environments, thus placing it a step closer to be deployed to a robot operating in the real world.

Furthermore, this system also demonstrated the role of feedback connections by showing preferential motor output for a particular object deemed more important. Although very limited in comparison with their extent in the brain, feedback connectivity plays in fact a major role in our model, by allowing the attention module to selectively inhibit neurons responding to the stimulus to be ignored, and thus allow the motor output to respond to the target only.

Considering the success of our hierarchical and modular design, we consider several enhancements and expansions to our network architecture. Similarly to the work of [20], [24], this modular design also allows us to extend our model in terms of scale and granularity of processing in the shape categorization module. While the current implementation of the shape categorization module considers only a single processing scale and two edges of orientation in the S1 layer (corresponding to V1 in the visual cortex), adding additional S1 cells with preference for alternate orientations or edge scales is a relatively simple task. Such additions would also allow us to extend the size of the visual field environment, learn more visual features, recognize more objects, and ultimately perform even more complicated recognition tasks. Further extensions would consider giving the system the ability to learn rotational invariance for visual stimulus. This could in principle be obtained by having the network learn to recognize the same object at different degrees of rotation and then associating its various representations at the classifier level via supervised learning.

In this paper, we considered the hardware constraints imposed by a digital neuromorphic hardware design [8], [9]. However, it is also important to point out that similar (though analog circuit) designs already are utilizing fabrication techniques to develop chips that consist of many cores [80]. Within a single fabricated wafer, a network of up to 180,000 neurons can be configured [80]. The most critical step in scalability lies therefore not in neuromorphic hardware resource availability, but in the possibility to easily design and deploy a large-scale network architecture. This could be done only with a modular architecture, to be designed with tools such as a hardware description language. In our implementation of an HMAX-like architecture we tried in fact to maximize modularity–and therefore scalability. For example the model could be expanded with the capability to recognize segments in an additional direction (i.e. not only vertical or horizontal) by adding a third set of layers (similar, for example, to S1-hor, C1-hor-ex, C1-hor-inh, C1-hor-max), which would differ from those already present only for they connectivity to the retina. Furthermore, inter- and intra-layer connectivity is also modular–since connectivity between two neurons only depends on their relative positions –and can be easily expanded for large retinas. Additional functionalities could be in principle implemented by adding additional layers on top of those already present. We will investigate more in detail these issues in future projects.

Beyond the shape categorization module, we also consider integrating a color processing module in future extensions to our neuromorphic system. The attentional system could also be enhanced by a color processing module. For example, areas of high contrast in the retina would likely be important, and the attentional system could direct the shape categorization module to specifically attend to such areas, as opposed to fully processing the entire visual field. Such extensions would both reduce the computational power required to robustly process visual information, as well as enhance the performance of the system significantly.

The learning algorithm employed here is based on biological plastic mechanisms. In related work [50], we were able to replicate several experimental findings using an NMDA-receptor dependent STDP algorithm with sleep-dependent renormalization. Burst-STDP is the translation of such a biological mechanism for networks of artificial spiking neurons, and here we proved its effectiveness for general learning tasks. Thus, these simulations provide a proof of principles on which burst-STDP is based: the metabolic cost strictly regulates the production and timing of spiking activity. Therefore, high firing rates must necessarily carry high information and must affect synapses more than sparse firing activity.

This paper has outlined the abilities of our network architecture, and we consider that, strikingly, all these tasks were achieved with a single elementary unit, the LIF neuron. While some layers were specifically organized in terms of topography or hard-wired connections, the entire system was built using a simplified model neuron with a limited number of configurable parameters. The modular architecture and configurable neuron model we have described are suitable for inexpensive hardware implementations as described in [8], [9], especially considering that homeostatic renormalization, which is key to the learning algorithm, naturally converges synapses to binary solutions. Aside from the number of neurons implemented (1000 in our network architecture, as opposed to 256 on the neuromorphic chips from [8], [9]), it is clear how neural networks of this design are primary candidates for deployment on energy efficient neuromorphic hardware designs in silicon.

In future work, we will consider fundamental comparisons between burst-STDP with homeostatic renormalization and other biologically inspired learning rules. Such a comparison would be quite useful, considering at least one neuromorphic chip exhibits online plasticity [9] with the choice of one of four learning rules: Hebbian, anti-Hebbian, STDP, and anti-STDP learning.
